# The Immune Microenvironment in Gastric Cancer: Prognostic Prediction

**DOI:** 10.3389/fonc.2022.836389

**Published:** 2022-04-28

**Authors:** Mingwei Ma, Juan Sun, Zhen Liu, Siwen Ouyang, Zimu Zhang, Ziyang Zeng, Jie Li, Weiming Kang

**Affiliations:** ^1^Chinese Academy of Medical Sciences and Peking Union Medical College, Beijing, China; ^2^Department of General Surgery, Peking Union Medical College Hospital, Beijing, China

**Keywords:** immune microenvironment, gastric cancer, prediction, CAF, TIL, TAM

## Abstract

Although therapeutic methods have been developed, gastric cancer (GC) still leads to high rates of mortality and morbidity and is the fourth leading cause of cancer-associated death and the fifth most common cancer worldwide. To understand the factors associated with the prognostic prediction of GC and to discover efficient therapeutic targets, previous studies on tumour pathogenesis have mainly focused on the cancer cells themselves; in recent years, a large number of studies have shown that cancer invasion and metastasis are the results of coevolution between cancer cells and the microenvironment. It seems that studies on the tumour microenvironment could help in prognostic prediction and identify potential targets for treating GC. In this review, we mainly introduce the research progress for prognostic prediction and the immune microenvironment in GC in recent years, focusing on cancer-associated fibroblasts (CAFs), tumour-associated macrophages (TAMs), and tumour-infiltrating lymphocytes (TILs) in GC, and discuss the possibility of new therapeutic targets for GC.

## Introduction

According to GLOBOCAN 2020, gastric cancer (GC) ranking fifth for incidence and fourth for mortality globally (1). The incidence rate and mortality of GC are higher in East Asia than in Europe (1), and the long-term survival rate of local advanced gastric cancer (LAGC) patients after surgery is less than 20%-30%. Although the survival time of GC patients can be improved by chemotherapy, the prognosis of LAGC is still poor even when treated with sequential lines of chemotherapy (2). Therefore, mining reliable indicators to predict the progression and prognosis of GC is urgently needed.

Immunotherapy has gradually appeared in the treatment of GC in recent years. Various types of immunotherapeutic approaches have been developed, such as vaccine therapy (3), chimeric antigen receptor (CAR) T cells (4), programmed cell death 1 (PD-1) and programmed cell death ligand-1 (PD-L1) (5–7). Despite these unprecedented anticancer clinical successes, the immunotherapy remission rates of various types of cancers remain low. In most clinical trials, immune checkpoint inhibitors failed to provide benefit in gastric cancer patients, although there were a few clinical trials showing that immunotherapy improves survival in selected GC patients (8). Therefore, the application of immunotherapy in GC also needs further investigation (9). Moreover, immune tolerance is obtained after receiving immunotherapy, mainly due to cross-talk between tumorigenesis and the immune response (10). Therefore, studies on the immune microenvironment in GC are of essential importance**(** 11**)**.

In recent years, many studies have shown that cancer invasion and metastasis are caused by interactions between cancer cells and the immune microenvironment (12–14). The tumour microenvironment (TME) plays an essential role in the process of tumour invasion and metastasis. Many studies have shown that the evolutionary mechanism of the TME is one of the critical reasons for the complexity, invasion, metastasis, and poor prognosis of GC (15, 16). Mesenchymal cells in the TME can significantly promote the invasiveness of cancer cells and become a new target in antitumour strategies (13), among which the immune microenvironment is critical. In the recruitment of tumour-related signals, various immune cell components infiltrate the immune microenvironment, interact closely with cancer cells, and then interact with each other to promote tumour development together (12, 14). Therefore, the new point of view is to fully understand the internal phase of the tumour immune microenvironment (17). Studies of the effect of TME components on cancer cells will help uncover new prognostic factors and potential therapeutic targets of GC (18).

**Figure 1:**
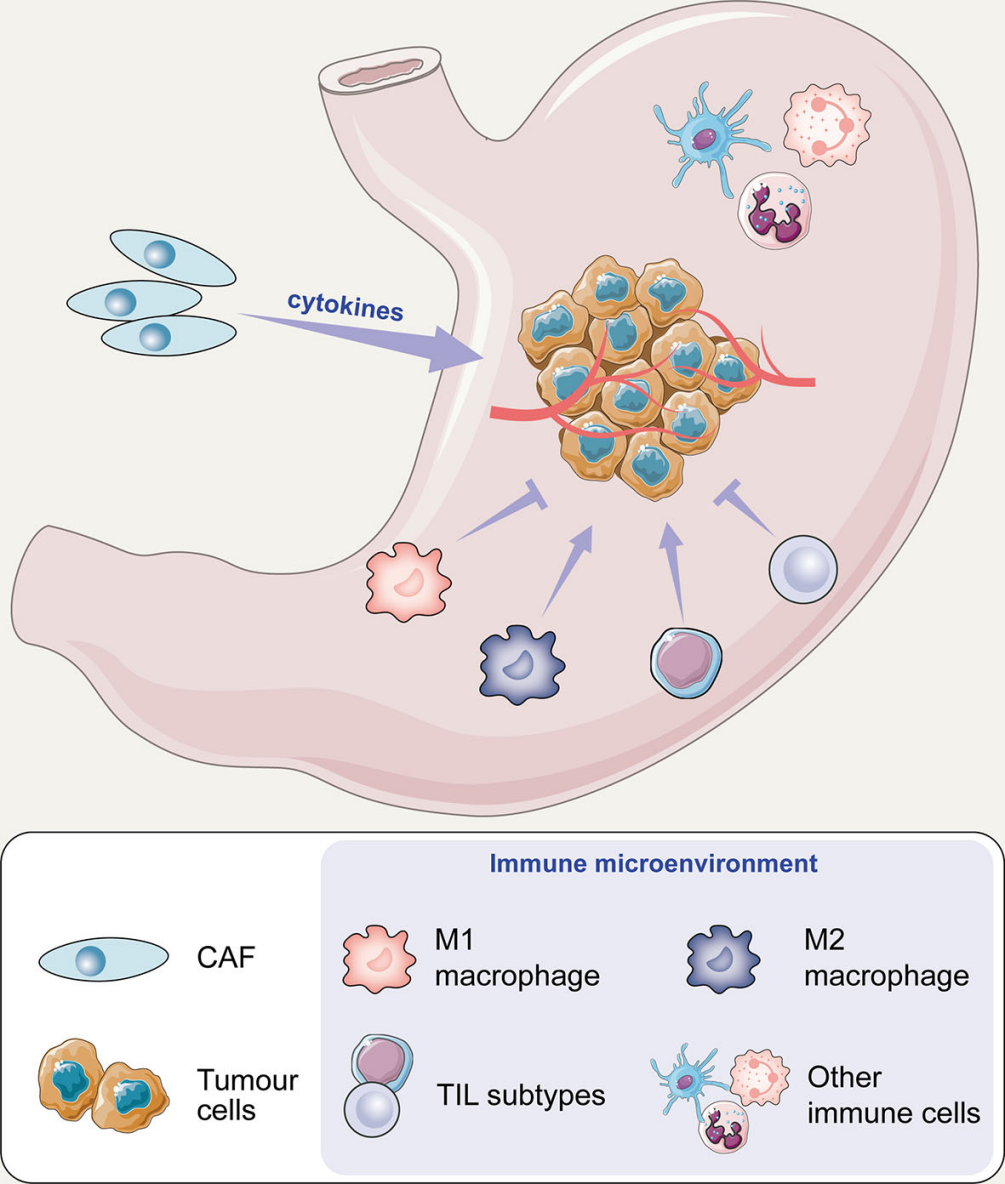
The potential mechanism of cell action on tumours in the immune microenvironment of gastric cancer.

**Figure 2:**
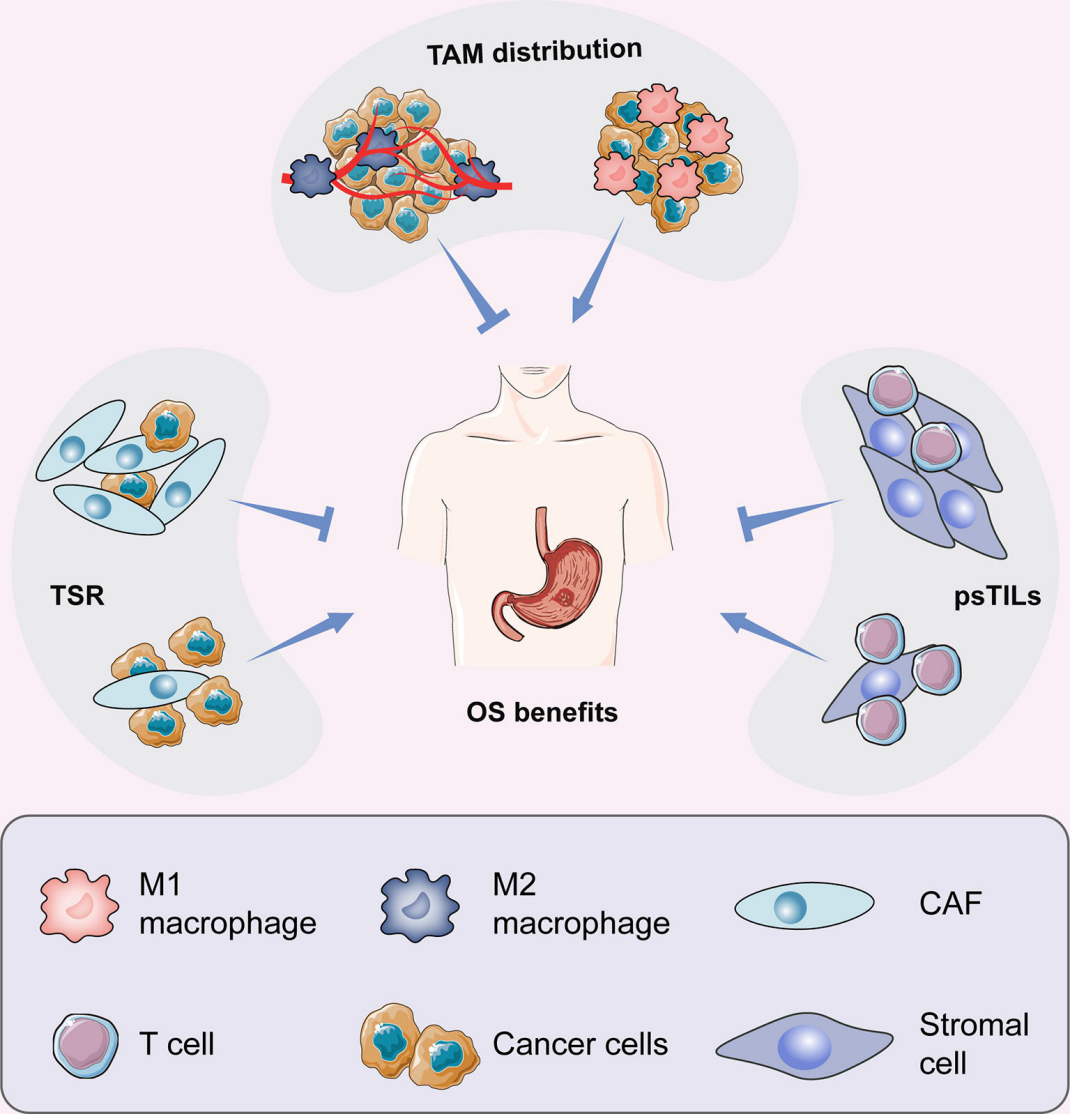
Prognostic prediction for CAFs, TAMs, and TILs in GC.

In this article, we reviewed the main components of stromal cells in the TME framework, including tumour-associated macrophages (TAMs), cancer-associated fibroblasts (CAFs), and tumour-infiltrating lymphocytes (TILs), which are involved in the regulation of the immune microenvironment (**Figure 1**). After understanding the interaction between cancer cells and the microenvironment, new antitumour methods targeting these cells may be gradually applied in clinical practice and will help in the design of individual-oriented therapy strategies for GC patients (**Table 1** and **Figure 2**).

**Table 1:** Functions of different cell types in gastric cancer.

Cell types	Functions in gastric cancer	References
Cancer-associated fibroblasts (CAFs)	Lymph node metastasis and tumour stage	(19–25)
	Promote tumorigenesis and development	(26, 27)
	Promote tumour progression	(28–30)
	Promote invasion and metastasis	(31, 32)
Tumour-infiltrating lymphocyte (TILs)	Potential prognostic value	(14, 33–39)
	Significance of TILs in tumour stroma is greater than that in tumour nest	(40)
	Different subtypes have different prognostic correlations	(36, 38, 41–44)
	High TIL density may have a bidirectional regulatory effect.	(45–47)
	Low psTIL group predicts poor overall survival	(48–50)
Tumour- associated macrophages (TAMs)	Proliferation, invasion and metastasis of cancer cells	(51–56)
	The number and type of TAMs affect the tumorigenesis and development	(17, 18, 35, 57–59)
	More TAMs predict a worse prognosis. Numerous TAM infiltrates suggests a good prognosis	(15, 60–69)
	TAMs at the tumour-mesenchymal junction affect tumour invasion	(70–72)
	TAMs promote angiogenesis in areas of vascular deficiency, necrosis and hypoxia	(73, 74)
	M2 macrophages predict a poor prognosis	(75, 76)

## Cancer-Associated Fibroblasts (CAFs)

Cancer-associated fibroblasts (CAFs), also called stromal cells, work as an essential component of the tumour microenvironment and perform diverse functions, including extracellular matrix deposition and tissue remodelling, cross-talk signalling, and interacting with cancer cells and infiltrating immune cells (77, 78).

Recent studies have shown that morphological analysis of the tumour-stromal ratio (TSR) at the periphery of tumour invasion can further evaluate the clinical significance of the TME and help in the discovery of new tumour prognostic indicators. As a new prognostic indicator, there is a significant positive correlation between high TSR, oesophageal cancer, breast cancer, and cervical cancer (21, 23, 24). In addition, a large amount of data shows that pathological image analysis based on HE staining and in-depth mining of TSR data information in tumour tissue can be used to a certain extent to improve the existing prognosis prediction system (20). Studies have reported that postoperative pathology needs to be evaluated for TSR indicators as an important supplementary note for tumour staging (25).

TSR is an important prognostic index, but it is still necessary to further analyse whether TSR parameters can represent the overall characteristics of the TME. According to the TME theory, it can be hypothesized that the more frequent the interaction is between the tumour and the interstitium, the greater the intensity of the action, the more significant the proliferation of various cellular components in the interstitium, and the greater the TSR value at the histological level. Previous studies have supported this conjecture that tumour cells can activate nearby tumour stroma, thereby promoting the evolution of the TME; activated tumour stroma components receive tumour-related signals and change both in quantity and morphology and then promote the occurrence and development of tumours (26, 27). The increase in the TSR value and the interstitial ratio will promote cancer progression, suggesting a poor prognosis. Regarding an explanation for this conclusion, the research shows that the main reasons include the following explanations: first, the increase in the interstitial ratio will release more interstitial-derived growth factors and other components, which will aggravate the tumour burden (28, 79); second, the tumour interstitial fibrosis can protect cancer nests by wrapping cancer cells to inhibit the body’s immune system from killing the tumour. The higher the interstitial ratio, the greater is the degree of fibrosis (11, 30); more importantly, the TSR value increases, implying that a small number of cancer cells can activate a large number of surrounding mesenchyme, which indicates that cancer cells, in this case, are more aggressive, and the cancer-interstitial interaction is more prominent, often resulting in a worse prognosis.

In addition, some studies have shown that when the proportion of stromal cells in some tumours is increased, the prognosis is often worse (31). The cancer cells in this part of the lesion transform and reshape the tumour microenvironment and have a greater ability to promote invasion and metastasis (31, 32). Therefore, an independent prognostic factor (TSR) of advanced GC has potential clinical practical value. GC assessment has the advantages of rapid operation, simplicity, and high repeatability at the methodological level; second, gastric cancer TSR has clinical relevance and prognostic value, indicating that the TSR is a prognostic indicator in the microenvironment. Studies have shown that Cohen’s kappa coefficient, a measure inter-rater reliability, is significantly increased in tumours, such as breast (19, 23), oesophageal (24), cervical (21), and colorectal cancers (20, 22, 25). The application value of these commonly used coefficients is similar to that of the TSR, which confirms that the TSR may be used as one of the routine parameters of clinicopathological analysis.

## Tumour-Infiltrating Lymphocytes (TILs)

Tumour-infiltrating lymphocytes (TILs) play an essential role in modulating the occurrence and development of tumours (80). In a phase 2 trial of KEYNOTE-158 (ClinicalTrials.gov identifier NCT02628067) (81), 24 patients with gastric cancer were treated with the PD1 humanized monoclonal antibody, pembrolizumab, and 11 patients responses and a median progression-free survival is 11 months. Notably, 4 patients with complete remission were included. The trial ultimately led to the U.S. Food and Drug Administration (FDA) approval of pembrolizumab for patients with unresectable or metastatic MSI-H or dMMR of any solid tumor type, including gastric cancer (82). Pembrolizumab inhibits PD-1 activity by binding to the PD-1 receptor on T cells and blocking the PD-1 inhibitory pathway, leading to T cell activation that inhibits tumor progression in GC patients. This result demonstrates the important role of T cells in tumor progression in patients with gastric cancer.

Under the recruiting influence of tumour signals, the appearance of mononuclear immune TILs infiltrating the tumour tissue can reflect the strength of the body’s antitumour immune response. Analysing the characteristics of TILs helps in clinical practice (34). Studies have shown that lymphocyte infiltration is a vital indicator of tumour progression (14). Subsequent to tumour chemotactic signalling, TILs gather in or around the cancer nest, which directly or indirectly promotes the invasiveness of cancer cells and ultimately affects prognosis (13). Clinical studies have shown that TILs have potential prognostic value in some tumours, including colorectal cancer (35), non-small-cell lung cancer (33), and other cancers (37). The cellular components of TILs are complex and have different functions. There are multiple subtypes, such as CD8+ T cells (38), CD4+ T cells (36), and B cells (43). In general, the overall extent of TILs has prognostic significance for tumours.

In addition, according to the latest consensus of the International TIL Working Group, the study of TILs in cancer nests is of little significance, and TILs in the tumour stroma need to be analysed. According to the consensus, it is appropriate to use the percentage of stromal TILs (psTILs) to study the degree of TIL infiltration in the interstitium rather than the number of TILs or the TIL density in the interstitium (40). Currently, the prognostic value of TILs in GC is still undefined (47). There is no consensus on whether TILs are protective or inhibitory factors. In addition, different GC TIL studies adopt different research protocols and cannot be compared with each other (83). The therapeutic regimen of TILs in GC needs to be further improved.

Some studies have explored the value of the percentage of stromal tumour-infiltrating lymphocytes (psTIL) as an indicator in GC, focusing on analysing the main clinicopathological characteristics of psTILs and the prognostic significance in GC patients. In theory, the specific cellular components of TILs have multiple subtypes, which can be divided into different cell types according to different antigens on the cell surface, leading to the complexity and changes in TIL research and significant differences in the interpretation of the results (36, 38, 43). Therefore, research on TILs has to initially solve the problem of methodological comparability. Currently, researchers investigating TILs mostly use immunohistochemistry to analyse the abundance and mechanism of different cell subtypes (41, 42). Whether TILs could become more acceptable has not yet reached a consensus. In addition, the prognostic correlation of different TIL subtypes varies, and some may even be antagonistic (44). A meta-analysis that included 4,185 cases of GC was conducted on the prognostic value of different subtypes of TILs in GC, but the results still suggested that the prognostic value of the different subtypes of TILs in GC is different. In addition, some studies have shown that a high proportion of TIL infiltration suggests that tumour patients have a longer survival time (46), which is contradictory. Another study suggests that high-density TILs may indicate a worse prognosis or a two-way regulatory effect (45). Due to different research methods, research objects (TIL subtypes), different TIL standards, and various other reasons, the existing research makes it challenging to intricately explore the specific value of TILs. Future studies can use HE staining to analyse the clinical significance of the ratio of TILs to the general interstitial area of GC (40). A randomized controlled clinical study (48) proved that psTILs have guiding significance in GC. The risk of GC in the low-value group of psTILs was significantly increased, the TNM staging was later, and the overall survival was worse.

In the process of tumour development, cancer cells interact with TILs and coevolve together. TILs exert their immune function to kill cancer cells; in contrast, upon induction by cancer cells, TILs may develop into different numbers or proportions of subtypes. When the antitumour-related subtypes decrease and the proportion of tumour-promoting subtypes increase, TILs become associated with a cancer-promoting effect (40). In GC research, cancer nests recruited TILs after EBV infection that were observed to be cytotoxic, and hence, the high psTILs indicated a better prognosis (84). However, in recent years, some studies have shown that some TIL subtypes, such as CD4+ T cells (CD4+ regulatory T cells, Tregs), may be one of the main factors that have an immunosuppressive role within the tumor (63). These Tregs appear late in GC, and presumable are a bad prognostic marker. Therefore, psTILs may have different prognostic values in different stages of GC. Incorporating psTILs into the prognostic model of GC may have specific guiding significance for the clinical diagnosis and treatment of GC. For example, GC with low psTILs may have a relatively poor prognosis. Such patients may benefit from enhanced clinical treatments, such as immunotherapy (29). More large-sample studies are still needed in the future to further confirm the clinical utility of psTIL parameters. In conclusion, psTILs show promise as an independent prognostic factor in GC, but greater clarity will be required concerning sub-populations and the context of their occurrence within the tumor microenvironment and within specific tumors.

## Tumour-Associated Macrophages (TAMs)

In the process of GC occurrence and development, the tumour microenvironment undergoes dynamic and complex changes, including tumour-related inflammation and angiogenesis (9). A key mechanism is that tumour cells release signals to recruit many immune-inflammatory cells. The functions of macrophages near the nest are complex and changeable (53, 55). There are a wide range of sources of macrophages, which can be derived from blood vessels and lymphatic vessels, as well as primary macrophages from the tumour, that can migrate to cancer nests and nearby macrophages to subsequently become tumour-associated macrophages (TAMs). In different parts of the cancer nest and mesenchyme, TAMs can produce and release various growth factors or chemokines to the surrounding area and participate in regulating cancer cell proliferation, invasion, and metastasis (52). Macrophage cells undergo phenotypic transformation, usually from the M1 type to the M2 type. The outcome of this transformation is to promote tumour progression; that is, a tumour suppressor-carcinogenic change occurs (85). Therefore, the role of TAMs is dual in nature.

In addition, studies have shown that the prognostic significance of TAMs for different tumour patients is also varied (51, 54, 56). It is believed that factors such as the number and type of TAMs can affect the various stages of tumour occurrence and development (35). 57 in 2003 used tumour-associated macrophage infiltration as one of the prognostic indicators of GC (57). Subsequently, many studies (17, 18)began to pay attention to the value of TAMs on the prognosis of GC and began to explore the relationship between TAMs and the clinicopathological characteristics of patients with GC. Some studies have shown that the higher the number of TAMs, the worse is the prognosis of GC (65, 66). However, other studies have shown that TAM infiltration in tumours can indicate a better prognosis (15). Therefore, the use of TAMs as an indicator to evaluate the prognosis of GC patients is still controversial. TAM indicators play an essential but two-way role in the prognosis of GC (60, 64).

The relationship between the total number of TAMs and the prognosis of GC patients has different conclusions. Studies have shown that the number of infiltrating macrophages in GC tissues is significantly higher than that in adjacent tissues, suggesting that as GC develops, macrophages are recruited and interact closely with cancer cells (86). Zhang et al. (87) used meta-analysis to evaluate the relationship between TAMs and solid tumours including GC-related studies. The quality of the included studies was not evaluated. Other studies have also found contradictions in the prognostic value of the total TAMs (61, 63). Some studies have shown that TAMs can promote tumours by inducing neovascularization and inhibiting the immune killing effect of the body (62), and other studies have suggested that TAM infiltration suggests a better prognosis for GC (15). Therefore, simply analysing the number of TAMs in GC research is insufficient to establish a new prognostic prediction model (88).

To solve the shortcomings regarding the number of TAMs, future research should propose a definition for the TAM distribution types, that is, according to the potential functions of TAMs in different locations, provide a comprehensive comparison, and analysis of different types of macrophages, and explore classifications that may be able to reflect, at least to a certain extent, whether TAMs participate in the evolution of GC-interstitial interactions (89). It has been reported that TAMs are more likely to undergo phenotypic transformation in the area where the tumour stroma is fully functional (90). On the one hand, tumour-associated macrophages can phagocytose and kill cancer cells and exhibit an antitumour effect; on the other hand, after interactions with cancer cells, TAMs may undergo a phenotypic change, such as M1 macrophages transforming into M2 macrophages (91); the former promotes the inflammatory response, which usually has an anti-tumour effect (92), while on the contrary, M2 macrophages enable the immune escape of cancer cells in the extracellular matrix. The remodelling and transformation of tumours and tumour angiogenesis are related, which will promote tumour development (93). In different microenvironments, TAMs function differently. For example, at tumour invasion sites, TAMs can promote the migration and invasion of tumour cells; in tumour stroma and tumour blood vessels, TAMs promote cancer metastasis; and in areas lacking blood vessels and areas of necrotic hypoxia, TAMs can promote angiogenesis (73). Therefore, the prognostic value of TAMs at different locations in GC tissues is different (74). Among them, the frontier of tumour invasion is a unique area. Recruited TAMs interact closely with cancer cells near the cancer nest and receive the most direct effect from the cytokines released by the interstitial components. They may actively participate in various signalling pathways (94) and continue to enhance their expression. Type transformation (95), ultimately the most critical factor, promotes the immune escape of cancer cells, the formation of new blood vessels, and the remodelling of the extracellular matrix (96). In addition, studies have found that M2-type macrophages are mainly recruited at the junction of cancer nests and stroma (75). In addition, there are also studies showing that the presence of a large number of M2-type macrophages at this location suggests that the prognosis of cancer patients is worse (75, 76). In summary, compared with simply analysing the total number of TAMs, by improving the research strategy, it is suggested that the TAM distribution type can be used as an important prognostic indicator for GC patients and included in the prognostic prediction model of GC.

## Other Immune Cells in GC

In addition to the widely studied T cells and macrophages, other immune cells infiltrate the tumour immune microenvironment in GC.

Natural killer cells (NK cells) are natural immune cells with the ability to kill tumour cells (97). Statistical analysis of clinical data shows a significant negative correlation between the percentage of NK cells in the tumour and the TNM stage of nerve-invaded tumours in patients with GC, suggesting that NK cells are closely related to GC progression (98). Some monocytes can upregulate the expression of CD69 in NK cells but significantly inhibit the expression of TRAIL, Ki-67, perforin, IFN-γ, and TNF-α. These results suggest that tumour-activated monocytes inhibit the function of NK cells (99).

Mast cells may promote tumour progression in the GC microenvironment. Mast cells can synthesize and release various growth factors and proteases(VEGF-A and MMP-9), thus promoting blood vessels and lymphatic generation (100). Studies have shown that mast cells in GC infiltration, which can promote angiogenesis and tumour lymph node metastasis, are related to poorer survival outcomes for patients with GC (101).

Immunosuppression is a significant feature of advanced GC and is closely related to GC progression. Previous studies have suggested that IL-35 is secreted by regulatory T cells, while recent studies have found that IL-35 can also be produced by B cells in mice and GC patients, and the expression of IL-35 in B cells is significantly upregulated in patients with advanced GC (102). In addition, the expression of IL-35 is positively correlated with other immune suppression factors, such as Treg cell infiltration and IL-10 expression, which indicates a poor prognosis.

## Conclusion

GC is a malignant tumor with high morbidity and mortality. At present, immunotherapy is applied for tumor treatment, but in GC, there are few clinical trials showing that immunotherapy can benefit patients. Patients with PD-L1 combined positive score (CPS) ≥5 who received nivolumab (PD-L1 inhibitor) plus chemotherapy improved OS compared with chemotherapy alone (103). However, the PD-L1 inhibitor avelumab did not prolong patient survival compared with the clinician’s choice of third-line therapy (104). More research should focus on the improvement and development of new immunotherapies.

The tumor immune microenvironment plays an important role in tumor progression and prognosis in patients with gastric cancer, and is also related to the response to immunotherapy. This article mainly reviews cancer-associated fibroblasts (CAFs), tumor-associated macrophages (TAMs), and tumor-infiltrating lymphocytes (TILs) in GC. Under the recruitment of tumor-related signals, various immune cell components in the immune microenvironment interact closely with cancer cells, and then evolve with each other to jointly promote the development of tumors. Cancer-associated fibroblasts (CAFs) can interact with tumor cells and immune cells. For example, CAFs can promote the invasive ability of cancer cells, and CAFs can also attract immune cell infiltration, including T cells and macrophages, by secreting cytokines and chemokines. Tumour-stromal ratio (TSR) may be regarded as one of the routine parameters of clinicopathological analysis. Tumor infiltrating lymphocytes (TIL) and tumor associated macrophages (TAM) are particularly important, but TIL and TAM is a double-edged sword in the immune microenvironment of gastric cancer, and plays a bidirectional regulatory role in the occurrence and development of gastric cancer. Therefore, the absolute cell number of TILs and TAMs couldn’t work as prognostic indicators. Increasing evidences suggested that psTILs are an independent prognostic factor of GC, and TAM distribution type can be used as an important prognostic indicator for GC patients. Among them, TILs may interact with TAMs, for example, the CD4 T cell-secreted IL4 may be involved in the M1-M2 transition (105), thereby facilitating tumor escape. The specific interaction mechanism among cancer-associated fibroblasts (CAFs), tumor-associated macrophages (TAMs), and tumor-infiltrating lymphocytes (TILs) remains to be further investigated. The current prognostic diagnostic factors and treatment methods also need to be further studied and improved. The combination of TSR, psTILs and TAM distribution type organically would help accurate prognostic prediction in GC in the future. The ultimate goal of GC prognosis prediction is to improve treatment of patients with GC who present at different stages.

## Author Contributions

MM and JS are the co-first authors of this paper. Other authors contributed to this writing and editing of this manuscript. All authors contributed to the article and approved the submitted version.

## Funding

This study was funded by the CSCO-ROCHE Research Fund (No. Y-2019 Roche-015), Beijing Xisike Clinical Oncology Research Foundation (Y-HS2019-43), Wu Jieping Medical Foundation (No. 320. 6750.19020, No. 320.6750.2020-08-32), CAMS Innovation Fund for Medical Sciences (2020-I2M-C &T-B-027) and Beijing Bethune Charitable Foundation (WCJZL202106).

## Conflict of Interest

The authors declare that the research was conducted in the absence of any commercial or financial relationships that could be construed as a potential conflict of interest.

## Publisher’s Note

All claims expressed in this article are solely those of the authors and do not necessarily represent those of their affiliated organizations, or those of the publisher, the editors and the reviewers. Any product that may be evaluated in this article, or claim that may be made by its manufacturer, is not guaranteed or endorsed by the publisher.
